# Simultaneous and delayed direct sinus lift versus conventional implants: Retrospective study with 5-years minimum follow-up

**DOI:** 10.4317/medoral.22612

**Published:** 2018-11-21

**Authors:** Javier Romero-Millán, Federico Hernández-Alfaro, Miguel Peñarrocha-Diago, David Soto-Peñaloza, David Peñarrocha-Oltra, María Peñarrocha-Diago

**Affiliations:** 1 Master in Oral Surgery and Implantology. Faculty of Medicine and Dentistry. University of Valencia, Valencia, Spain; 2 Department of Oral and Maxillofacial Surgery, School of Dentistry, Universitat Internacional de Catalunya, Barcelona, Spain; 3 Professor and Chairman of Oral Surgery, Stomatology Department, Faculty of Medicine and Dentistry, University of Valencia, Valencia, Spain; 4 Assistant Professor of Oral Surgery. Faculty of Medicine and Dentistry. University of Valencia, Valencia, Spain; 5 Full Professor of Oral Surgery. Faculty of Medicine and Dentistry. University of Valencia, Valencia, Spain

## Abstract

**Background:**

To compare the radiological parameters and success of posterior maxillary direct sinus lift with simultaneous or delayed implant placement, or implant placement in native bone, after a minimum follow-up period of 5 years.

**Material and Methods:**

A retrospective cohort study was carried out in a university clinic, selecting patients subjected to implant treatment in the posterior maxilla between the years 2005 and 2011. The patients were divided into three groups: 1) implants placed in native bone; 2) direct sinus lift with simultaneous implant placement; and 3) direct sinus lift with delayed implant placement. Bone crest level, bone loss, vertical bone gain, and implant success and survival after a minimum follow-up period of 5 years after prosthetic loading were analyzed.

**Results:**

A total of 163 patients and 329 implants were included in the study. The mean duration of follow-up was 7.0 ± 1.9 years. Bone loss and implant success and survival were very similar in all three groups, with no significant differences among them. Graft reabsorption was greatest during the first 12 months, though graft stabilization was confirmed after 5 years of follow-up.

**Conclusions:**

Bone loss and percentage success and survival proved very similar for the implants placed in native bone and for sinus lift with simultaneous or delayed implant placement. The height of the graft material decreased mainly in the first 12 months, and continued until stabilization after 5 years, with no significant variations thereafter.

** Key words:**Sinus lift, pristine bone, native bone, dental implants, marginal bone loss, radiological study, implant survival, implant success.

## Introduction

Implant insertion in the posterior maxilla can be problematic due to insufficient vertical and horizontal bone volume, and proximity to the maxillary sinus (1,2). In addition, bone quality is frequently unfavorable. The cancellous bone is often of low density (3,4). The sinus floor augmentation or sinus lift technique was developed to increase the vertical bone level in order to secure primary stability of endosseous implants (5).

Sinus augmentation has proven to be a safe procedure, with predictable outcomes (6,7). Nevertheless, the comparison of bone loss associated to dental implants placed in the posterior maxilla with or without sinus lift has yielded discrepant results in the literature. Johansson *et al.* (8) recorded practically the same bone loss in both groups (1.1 mm in the control group vs 1.4 mm in the sinus lift group). Galindo *et al.* (9) recorded greater bone loss in implants placed after direct sinus lift compared with implants placed in native bone (0.83 mm vs 1.20 mm), while in contrast Schlegel *et al.* (10) found 70.4% of the implants placed in native bone to present bone loss versus only 41.4% of the implants in the sinus lift group.

On analyzing the bone gained as a result the sinus lift procedure, discrepancies have likewise been reported in terms of graft behavior. In effect, while Zijderveld *et al.* (11) recorded a decrease in graft volume during the first year, followed by stabilization, Tetsch *et al.* (12) observed a decrease over the first 6 months, followed by an increase in graft area during the second half of the first postoperative year.

Few studies have compared treatment success according to whether sinus augmentation is performed or not. Most publications have focused on implant survival. The discrepancies in the radiological analyses of the different studies can also be extrapolated to success. In 2009, Sbordone *et al.* (13) recorded a success rate of 95.8% after three years for implants placed in native bone, versus 85% for implants placed after sinus lift with bone particle grafting, though the difference between the two groups was not significant. In contrast, Wannfors *et al.* (14) recorded a significantly greater success rate for implants placed in native bone.

Based on the existing scientific evidence, we hypothesized that implant placement in clinical areas affects the radiological outcomes. The aim of the present study was to compare the success rate and radiological peri-implant parameters (bone loss and vertical bone gain) of implants placed in the posterior maxilla in native bone versus sinus lift with simultaneous or delayed implant placement.

## Material and Methods

-Study design

A retrospective cohort study with a minimum follow-up period of 5 years was carried out. We included all patients consecutively rehabilitated with dental implants in the posterior maxilla (from the first premolar to the second molar) in the Oral Surgery Unit of the University of Valencia (Valencia, Spain) during the period 2005-2011. The study was carried out in abidance with the principles of the Declaration of Helsinki referred to clinical research in human subjects. Written informed consent was obtained from all patients, and the study was approved by the local Ethics Committee (Reference: H1410262226693).

-Study population

The study included patients subjected to direct sinus lift (DSL) with simultaneous or delayed implant placement, and patients with sufficient available bone to allow integral implant placement in native bone without the need for bone grafts.

Patients without radiographic evaluations (panoramic and periapical X-rays) on any of the control visits were excluded, as were patients referred from other centers, patients failing to come to any of the scheduled control visits, patients declining to participate in the study, and patients with a duration of follow-up of less than 5 years.

The patients were divided into three groups:

• Control group: Patients subjected to integral implant placement in native bone, without the need for any bone regeneration technique.

• Study group 1: Patients subjected to DSL with simultaneous implant placement.

• Study group 2: Patients subjected to DSL with delayed implant placement (after 6 months).

-Surgical procedure

All the operations were carried out by the same surgeon (MPD) in the operating room under local anesthesia with 4% articaine and 1:100,000 adrenalin (Inibsa®, Lliça de Vall, Barcelona, Spain). Phibo® TSA implants (Phibo Dental Solutions, S.L., Sentmenat, Barcelona, Spain) were used.

•Control group

The implants in this group were placed conventionally, following the drilling sequence recommended by the manufacturer.

•Study groups 1 and 2

The same direct sinus lift procedure was performed in both study groups. The window ostectomy was started with a round tungsten carbide drill and completed with ultrasound (Surgysonic®, Esacrom, Imola, Italy). Detachment of the Schneiderian membrane was carried out by combining ultrasound instruments with manual curettes. In all cases we used β-tricalcium phosphate (KeraOs®, Keramat S.L.U., Ames, A Coruña, Spain) as the only graft material, and the sinus window was covered with a reabsorbable collagen membrane (Bio-Gide®, Geistlich Pharma AG, Wolhusen, Switzerland). The implants were positioned following the drilling sequence recommended by the manufacturer in the same surgical step in study group 1 and in a second operation 6 months later in study group 2.

The following postoperative medication was prescribed in all cases: amoxicillin – clavulanic acid (Augmentine®, GlaxoSmithKline, S.A., Madrid, Spain) 500 mg/8 hours during 7 days; ibuprofen (Bexistar®, Laboratorio Barcino, Barcelona, Spain) 600 mg/8 hours during three days; and 0.12% chlorhexidine rinses (GUM®, John O. Butler Co., Chicago, IL, USA) three times a day during 7 days.

The healing caps were placed in a second surgical procedure after three months of healing in the control group and after 6 months in both direct sinus lift groups. The prostheses were prepared after approximately of 4 weeks.

-Follow-up and maintenance

All the patients underwent annual control visits in which professional cleaning was performed. At the time of the study, all the subjects had been followed-up on for a minimum of 5 years, but man had been follow-up for a longer period.

-Data collection

Bone loss, vertical bone gain, and implant success and survival were assessed at each timepoint during follow-up. Two time-points were used: 5-year follow-up (available in all cases) and maximum follow-up (which was heterogeneous, ranging from 5 to 12 years). For maximum follow-up, the last available data for each case was used for the analysis.

•Bone loss

**Figure 1:**
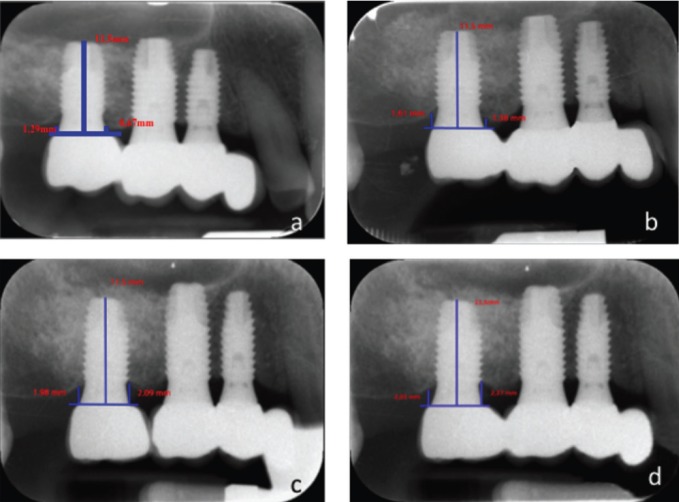
Periapical X rays showing the measurement of bone crest level both mesial and distal at the different study timepoints. Bone loss is recorded as the difference between the different moments over follow-up and prosthetic loading. a) Prosthetic loading; b) 12 months after loading; c) 5 years after loading; d) Maximum follow-up, 8 years after loading.

Bone loss was evaluated based on the method described by Boronat *et al.* (15). Radiological exploration was carried out with an XMIND intraoral system (Groupe Satelec-Pierre Rolland, Bordeaux, France) and a radiovisiographic (RVG) intraoral digital receptor (Kodak Dental System, Atlanta, GA, USA). To reproduce the patient alignments, a rigid cross-arch bar was used with bite-registration material, and a Rinn XCP (Dentsply, Des Plaines, IL, USA) rod and ring were firmly attached to the bar and placed in contact with the X-ray cone. The receptor was held by a slot in the bar. Software-based measurements were made (in mm) of implant marginal bone loss at the time of loading and on each of the control visits. For measurement purposes, two visible and easily localized reference points were selected at the junction point between the implant and the prosthetic restoration. A straight line was traced joining the two reference points and was considered to represent zero height. For the determination of bone loss, a perpendicular line was traced mesial and distal to the implant from zero height to contact with the bone. The difference between the value recorded at the time of loading and on each follow-up visit was used to calculate bone loss mesial and distal to the implant (Fig. 1).

•Vertical bone gain

**Figure 2:**
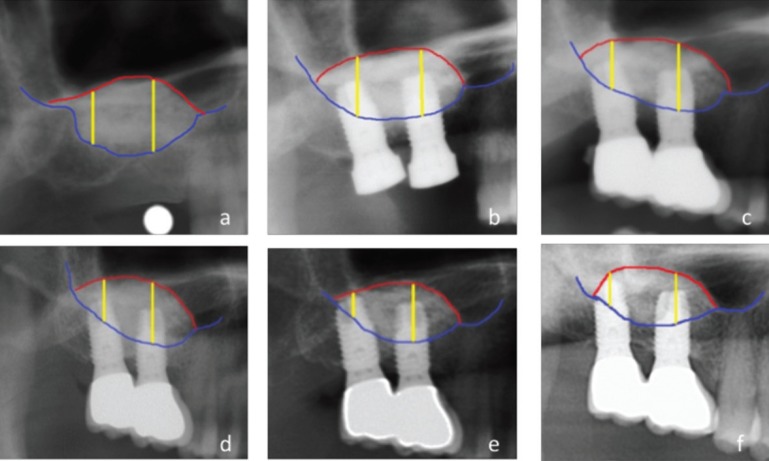
Panoramic X-ray measurements made to evaluate bone graft height at the different study timepoints. a) Sinus lift; b) Implant placement; c) Prosthetic loading; d) 12 months after loading; e) 5 years after loading; f) Maximum follow-up, 9 years after loading.

Panoramic X-rays were used to record vertical bone gain (Ortopantomograph® OP 100, Instrumentarium Imaging, Tuusula, Finland) as described by Peñarrocha *et al.* (16), using the Cliniview® version 5.1 application (Instrumentarium Imaging, Tuusula, Finland). The X-ray system was calibrated before the measurements were made: with the length of the implants in the case of simultaneous implant placement, and with a 5-mm steel ball in the case of delayed implant placement. We measured the height (in mm) from the lower sinus cortical layer to the upper limit of the graft material. In the case of delayed implant placement, the first bone graft measurement was made after surgery in the zone where the implant subsequently would be placed. In the case of implants already in place, the measurements were obtained at the center of the implant. We obtained as many measurements as there were implants placed (Fig. 2).

•Implant success and survival

The definition of success was based on the clinical and radiological criteria of Albrektsson *et al.* (17). Implant survival in turn was considered when the implant was present in the mouth and performing its function, regardless of its condition.

-Statistical analysis

A descriptive statistical analysis was made of the continuous (mean, standard deviation, median, minimum and maximum) and categorical variables (absolute frequency and percentage). With regard to the inferential analysis, we evaluated the homogeneity of the control group and the two DSL groups with regard to the patient parameters, the surgical variables, implant characteristics and clinical parameters using the Pearson chi-squared test, the Fisher exact test, the Student t-test and analysis of variance (ANOVA) – F statistic. Repeated measures unifactorial ANOVA was used to study the evolution of bone loss and vertical bone gain. Estimation of the survival and success rates was based on Kaplan-Meier models. The homogeneity of the survival functions in the different groups was evaluated using the log-rank test. The level of significance considered was 5% (α=0.05). All analyses were performed using the SPSS version 15 statistical package (SPSS, Chicago, IL, USA).

## Results

We reviewed 234 patients amenable to inclusion in the study. Of these, 28 were excluded because they had been referred from other centers and were not followed-up on in our clinic; 32 were excluded due to incomplete radiographic records; and 11 were excluded because they failed to report to the annual control visits. The final study sample thus comprised 163 patients and 329 implants. The length of the implants ranged from 8.5 mm to 16 mm and the diameter from 3.6 mm to 5.5 mm. No differences were found in the length or diameter of the implants between any of the groups (*p*>0.001).

The mean duration of follow-up was 7.0 ± 1.9 years. The number of patients in each group, together with age, gender, the number and position of implants placed, the form of implant healing (submerged/exposed), type of prosthesis, number of sinus lifts, basal bone height and follow-up are described in Table 1. Statistically significant differences were observed in the position, form of implant healing, type of prosthesis and basal bone height. None of the other variables showed statistically significant differences between groups ( Table 1).

**Table 1:**
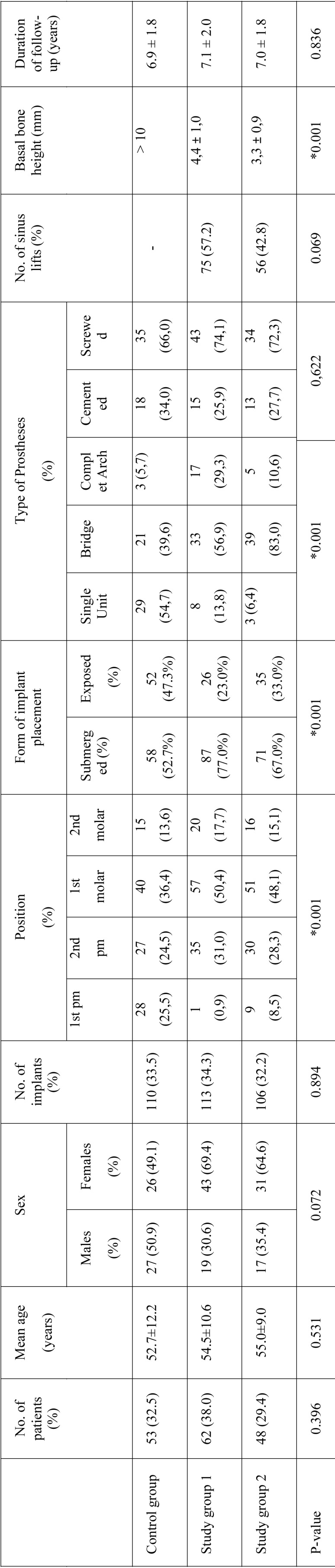
Study variables in the different groups.

-Bone loss

Bone loss is reported in  Table 2 for each of the groups.

**Table 2:**
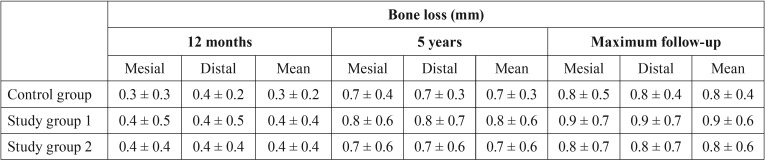
Bone loss recorded at each of the timepoints over follow-up.

After 12 months, bone loss in the control group was similar to that observed in study group 1 (*p*=0.329) and study group 2 (*p*=0.647). The difference between the two study groups likewise proved nonsignificant (*p*=1.000). After 5 years, bone loss in the control group was similar to that observed in study group 1 (*p*=0.198) and study group 2 (*p*=1.000). The difference between the two study groups likewise proved nonsignificant (*p*=0.441). On the last follow-up visit, bone loss in the control group was similar to that observed in study group 1 (*p*=0.296) and study group 2 (*p*=1.000). The difference between the two study groups likewise proved nonsignificant (*p*=0.371). The evolution of bone loss over time therefore can be regarded as similar in all three groups (*p*=0.507).

•Vertical bone gain 

The gain in maxillary vertical bone could only be analyzed in the case of maxillary direct sinus lift with simultaneous or delayed implant placement (study groups 1 and 2). We evaluated a total of 110 patients with 219 implants. The data corresponding to each of the measurement timepoints are shown in  Table 3. In general, a significant decrease in vertical bone gain was noted over time in the two study groups (*p*<0.001). Graft reabsorption was greatest in the first 12 months, though from 5 years of follow-up no statistically significant decrease was observed (*p*=0.458 and p=0.086, groups 1 and 2, respectively), thus evidencing stabilization of the bone graft. On the other hand, mean vertical bone gain was greater in the delayed implant group than in the simultaneous implant group at all timepoints (*p*<0.001). The evolution of vertical bone gain over time therefore can be regarded as similar in all three groups (*p*=0.630).

**Table 3:**
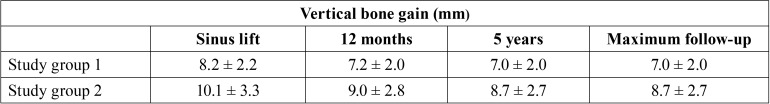
Maxillary vertical bone gain.

-Success and survival of the implants

**Figure 3:**
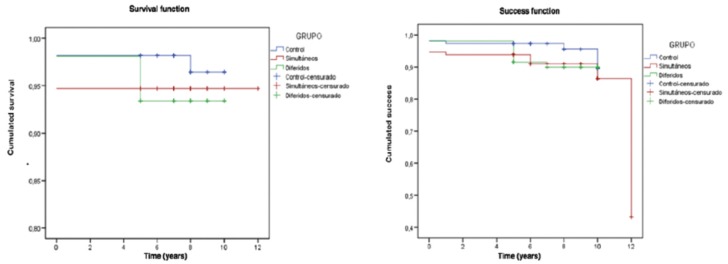
Kaplan-Meier success and survival rates curves.

Implant success and survival in each of the groups after 12 months and 5 years, and on the last follow-up visit, are described in  Table 4. No statistically significant differences were observed on comparing the cumulative success rates after 10 years among the three groups (*p*=0.297, log-rank test). On considering the 12 year timepoint, the success rate in the simultaneous implant group was very low (44.0%), because on the last follow-up visit we evaluated two implants, of which one presented problems. We likewise observed no statistically significant differences in cumulative survival among the three groups (*p*=0.405, log-rank test) (Fig. 3).

**Table 4:**
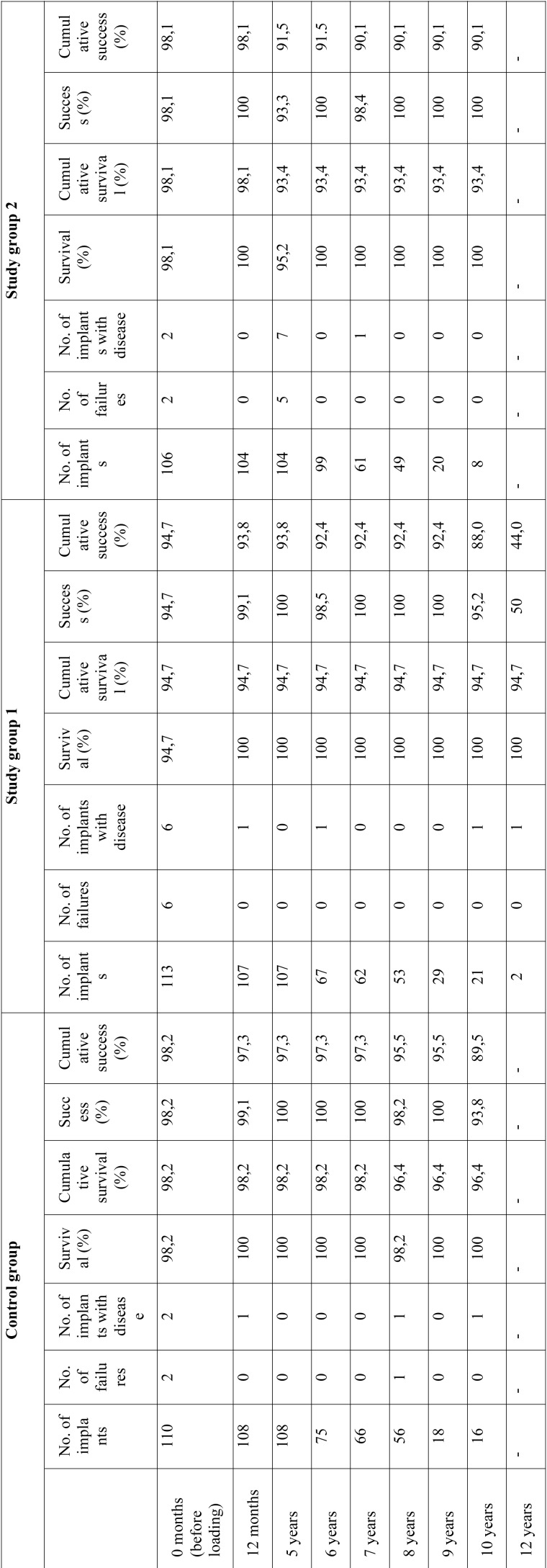
Analysis of implant success and survival rates of study groups.

## Discussion

Sinus augmentation with implant placement is the most widely used technique for rehabilitating the atrophic posterior maxilla. However, it is difficult to clearly define the long-term behavior of such implants, since the data found in the literature are contradictory. We therefore carried out a study involving a long follow-up period in order to analyze and compare the radiological parameters and success and survival rates associated to direct sinus lift with simultaneous or delayed implant placement, and implant placement in native bone.

The bone loss recorded in our study on occasion of the last follow-up visit was 0.8±0.4 mm in the control group, 0.9±0.6 mm in study group 1, and 0.8±0.6 mm in study group 2. There were no statistically significant differences among the groups at any of the analyzed timepoints, though significant and very similar bone losses were recorded throughout the follow-up period. These findings are consistent with those of most authors. Johansson *et al.* (8) placed 206 implants and recorded a mean bone loss after 36 months of 1.1 mm in the control group versus 1.4 mm in the sinus lift group. Sbordone *et al.* (13) in turn placed 70 implants and observed a bone loss of 1.1 mm in the control group and of 1.3 mm in the study group. In contrast, Galindo *et al.* (9) recorded significant differences; with greater bone loss in implants placed after direct sinus lift than in implants placed in native bone (0.83 versus 1.20 mm) after 36 months of follow-up. Only one of the analyzed studies obtained completely contradictory results. Schlegel *et al.* (10) placed 141 implants (71 in native bone and 70 after DSL), and at 1.6 years of follow-up found 70.4% of the implants placed in native bone to exhibit bone loss versus only 41.4% of the implants in the sinus augmentation group.

The maxillary vertical bone gain was determined from calibrated panoramic X-rays. The use of panoramic radiographic techniques could be regarded as a limitation, though they have been validated for this purpose (22,23). Cone beam computed tomography (CBCT) would afford greater measurement precision, but implies increased radiation exposure for the patient. Periapical X-rays in turn only afford small images and would not allow evaluation of the entire bone graft, in contrast to panoramic X-rays. Moreover, by using a standardized parallel technique, image reproducibility is ensured. In our study, the vertical bone gain at the time of surgery was 8.2 ± 2.2 mm in study group 1 and 10.1 ± 3.3 mm in study group 2. This height was seen to decrease significantly after 12 months, reaching 7.2 ± 2.0 mm in study group 1 and 9.0 ± 2.8 mm in study group 2. From this moment onwards the graft was seen to stabilize, since bone reabsorption practically ceased. As a result, bone height remained without statistically significant variations after both 5 years and on occasion of the last control visit.

Using the same measurement method, Sánchez-Recio *et al.* (24) and Peñarrocha *et al.* (16) recorded a mean gain in bone height of 7.2 mm and 6.7 mm, respectively. These figures are slightly lower than our own, though the mentioned authors only included sinus lift procedures with simultaneous implant placement. Furthermore, they only performed measurements at the time of surgery; as a result, we are unable to compare the graft volumetric changes in our series with those of the aforementioned studies. Nevertheless, a number of authors have analyzed such changes, and most of them obtained results consistent with our own (11,13,25,26). Hatano *et al.* (26) used panoramic X-rays at different timepoints, and after surgery found the new maxillary sinus floor to lie above the implant apex. After 2-3 years the floor was seen to have leveled with the apex or lie slightly below the apex. This situation in turn remained stable over time. Zijderveld *et al.* (11) recorded statistically significant graft reduction independently of the type of material used. Most of this reduction occurred in the first 1.5 years, after which the changes proved minimal, and the graft was seen to remain practically stable after 5 years. Only Tetsch *et al.* (12) recorded a decrease in the first 6 months, followed by an increase in graft area in the second half of the first postoperative year - resulting in restoration of the original graft volume.

The cumulative success rate in our study after 10 years of follow-up was 89.5% in the control group, 88.0% in study group 1, and 90.1% in study group 2. There were no statistically significant differences among the groups. Few studies have examined implant success. Sbordone *et al.* (13) recorded a three-year success rate of 95.8% for implants placed in native bone versus 100% for implants placed after sinus lift with particulate iliac crest, chin block or iliac crest block. The use of particulate chin bone lowered this figure to 85%, though without significant differences between the groups. In contrast, Wannfors *et al.* (14) obtained a significantly higher success rate for implants belonging to the control group (95.6%), with no statistically significant differences between the two sinus lift groups (79% in the simultaneous implant placement group and 89.2% in the delayed implant placement group). In contrast to implant success, survival has been analyzed in most studies. Huynh-Ba *et al.* (27), Uckan *et al.* (28) and Sbordone *et al.* (25) recorded survival rates of over 90% for both implants placed after sinus lift and implants placed in native bone, with no statistically significant differences between the groups. Other studies did record significant differences, however. Barone *et al.* (29) recorded a cumulative survival rate of 86.1% for implants placed after sinus lift versus 96.4% for implants placed in native bone. These differences were highly significant – thus indicating that implants placed after sinus lift are more likely to fail. Similar results were obtained by Sesma *et al.* (30), who found implants placed after sinus lift to be 5.5 times more likely to fail than implants placed in native bone.

Our study has limitations that make it necessary to view the results with caution. The main limitations of the study are its controlled but non-randomized retrospective cohort design, and the heterogeneity of the follow-up period of the patients beyond five years of loading. Nevertheless, this is the study with the largest sample and longest duration of follow-up comparing the success of implants positioned after sinus lift and in native bone. The next step will be to conduct a randomized, controlled prospective study and compare the results obtained with other posterior maxillary rehabilitation techniques.

## Conclusions

Bone loss and percentage success and survival proved very similar for implants placed in native bone and for sinus lift with simultaneous or delayed implant placement. The height of the graft material decreased mainly in the first 12 months and continued until stabilization after 5 years, with no further variations thereafter.
